# Bibliometric analysis of neonatal sepsis from 2002 to 2022

**DOI:** 10.1002/pdi3.49

**Published:** 2024-03-15

**Authors:** Chang Liu, Feifan Chen, Yuan Shi

**Affiliations:** ^1^ Department of Neonatology Children's Hospital of Chongqing Medical University Chongqing China; ^2^ National Clinical Research Center for Child Health and Disorders Chongqing China; ^3^ Ministry of Education Key Laboratory of Child Development and Disorders Chongqing China; ^4^ Chongqing Key Laboratory of Pediatrics Chongqing China

**Keywords:** bibliometric analysis, CiteSpace, neonatal sepsis, research hotspot, research trends

## Abstract

This study aimed to visualize the knowledge structure and research trends in neonatal sepsis research through bibliometric methods. Articles and reviews regarding neonatal sepsis from 2002 to 2022 were retrieved from the Web of Science Core Collection database. CiteSpace software was used to visualize the knowledge network of countries/regions, institutions, authors, journals, keywords, and references in this field. Altogether, 2314 publications were identified. During the study period, the number of publications increased yearly. The USA is the leading country in neonatal sepsis research. Duke University was the most prolific institution, with Pediatric Infectious Disease Journal and BASU S being the most prolific journal and author in the field, respectively. Pathogen, diagnosis, and management were the main topics of research, and future studies may concentrate on novel diagnostic biomarkers and judicious use of antibiotics. In summary, the results of our bibliometric analysis revealed views on the current situation and trends of neonatal sepsis research for the first time. This study may provide guidance for promoting research on neonatal sepsis.

## Graphical abstract

The USA and Duke University were the leading contributors in neonatal sepsis research. The Pediatric Infectious Disease Journal and author BASU S had the highest productivity. Research primarily focused on pathogens, diagnosis, and management. Future studies may prioritize novel diagnostic biomarkers and the appropriate use of antibiotics.

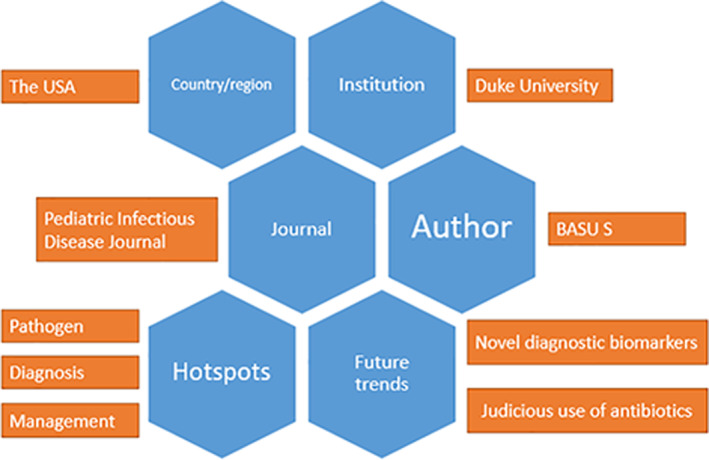

## 1 INTRODUCTION

Sepsis is defined as life‐threatening organ dysfunction caused by a dysregulated host response to infection.^1^ Newborns are one of the highest risk groups for sepsis, and neonatal sepsis is a first order public health system issue owing to its extremely high risk of mortality and morbidity.^2^ An epidemiological systematic review showed that the prevalence of neonatal sepsis varied across regions, with the global overall prevalence calculated to be 2202 per 100,000 live births, and the mortality ranged from 11% in the USA to 19% in India.^2^ Based on the age of the onset, neonatal sepsis can be divided into early onset sepsis (EOS) and late onset sepsis (LOS).^3^ Clinical manifestations of neonatal sepsis are variable, so any newborn with abnormal vital signs, sudden decline in feeding, or obvious changes in mental status, tone, or perfusion requires investigation for sepsis. The mainstay of treatment for neonatal sepsis is broad spectrum intravenous antibiotics, which will be narrowed once the pathogen and its antibiotic sensitivity are identified.^3^

Bibliometrics, a feasible method of statistical analysis, can be used to give a valuable overview of existing academic literature and predict the development trends of a given research subject based on published research.^4^ It has been widely applied in medical fields such as pulmonary arterial hypertension and myopia.^5^, ^6^

Studies on neonatal sepsis have sprung up over the last 20 years, with an enormous number of publications and different research directions; however, these studies failed to directly show the development, research hot spots, and future trends of neonatal sepsis research. In general, there is a lack of literature on the overall development context and research hot spots in the field of neonatal sepsis. Therefore, the objective of the study was to explore the knowledge structure, research hot spots, and potential trends on neonatal sepsis using a bibliometric analysis based on data from the Web of Science Core Collection (WoSCC) from 2002 to 2022.

## 2 METHODS

### 2.1 Sources of the data and search strategy

Data were extracted from the WoSCC electronic database (SCI‐Expanded), which is regarded as the most comprehensive and authoritative database platform for accessing global academic information and is widely used for bibliometric analysis.^5^, ^7^ We performed a systematic literature search on a single day (September 12, 2022) to prevent bias caused by daily database updates. Publications were limited to reviews and articles published in English between September 12, 2002 and the time of data collection. The search terms were as follows: TS = ([“sepsis”] OR [“early onset sepsis”] OR [“late onset sepsis”]) AND TS = ([“neonatal”] OR [“neonate*”] OR [“infant*”]).

### 2.2 Data extraction

Data extraction was conducted by two independent researchers to ensure relevance to the research topic. Any disagreements between the two researchers in the screening process were resolved through consultation between them until a consensus was reached. The data were selected with “Full record with cited references” and exported in “plain text file format.”

### 2.3 Data analysis

Microsoft Excel 2013 (Microsoft Corporation) software was used to show annual outputs, arrange and sort the data, and extract the top results. CiteSpace software (Version 6.1. R3, USA), one of the most popular software programs for bibliometric analysis, was applied to visualize the bibliometric data. Created by Prof. Chaomei Chen and based on the Java programming language, it can conduct collaborative network analysis, co‐occurrence analysis, and co‐citation analysis to reveal the collaborative networks of countries/regions, institutions, and authors, the distribution of subject areas, high‐frequency keywords, and core literature in a specific research area in the form of visual graphs or tables. VOSviewer (Version 1.6.18, USA) and Scimago Graphica (USA) were combined to visualize and analyze the cooperative relationships between countries/regions.

## 3 RESULTS

### 3.1 General description of retrieved publications and publication years

**FIGURE 1:**
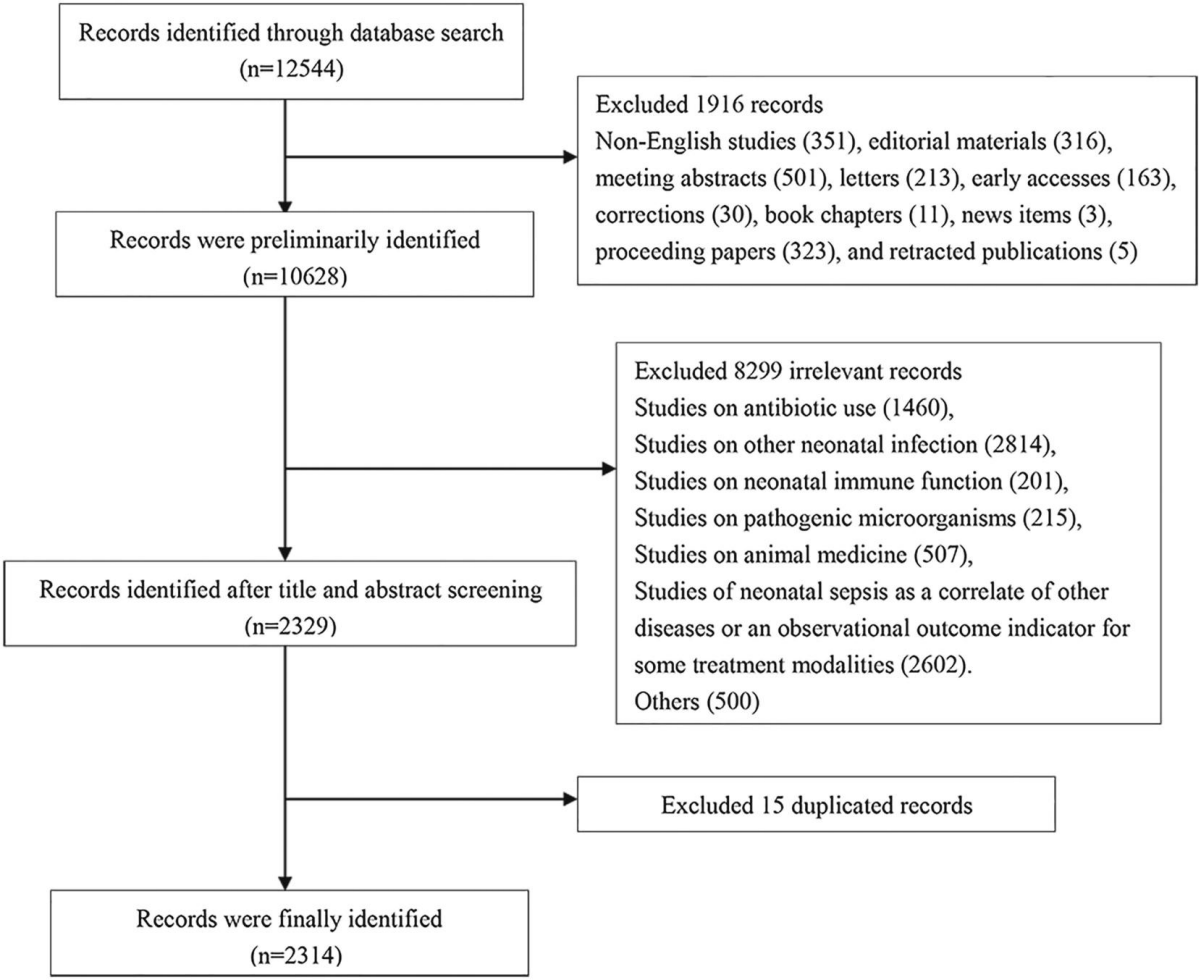
Flowchart of the literature search, screening, and analysis.

We retrieved a total of 12,544 documents on neonatal sepsis based on our search strategy from WoSCC at the time of data collection. We excluded non‐English studies (351), editorial materials (316), meeting abstracts (501), letters (213), early accesses (163), corrections (30), book chapter (11), news items (3), proceeding papers (323), and retracted publications (5). Next, we further performed title and abstract screening of all included literature, and 8299 publications in total were removed because they did not take neonatal sepsis as the main research content. Subsequently, the rest of these documents were imported into CiteSpace software, and 15 duplicates were excluded. Finally, 2314 studies were included in the analysis, containing 2012 articles and 302 reviews, and 2985 institutions from 106 countries/regions contributed to these publications. The flowchart of the study search and the screening process was given in Figure 1.

### 3.2 Annual publication trend

**FIGURE 2:**
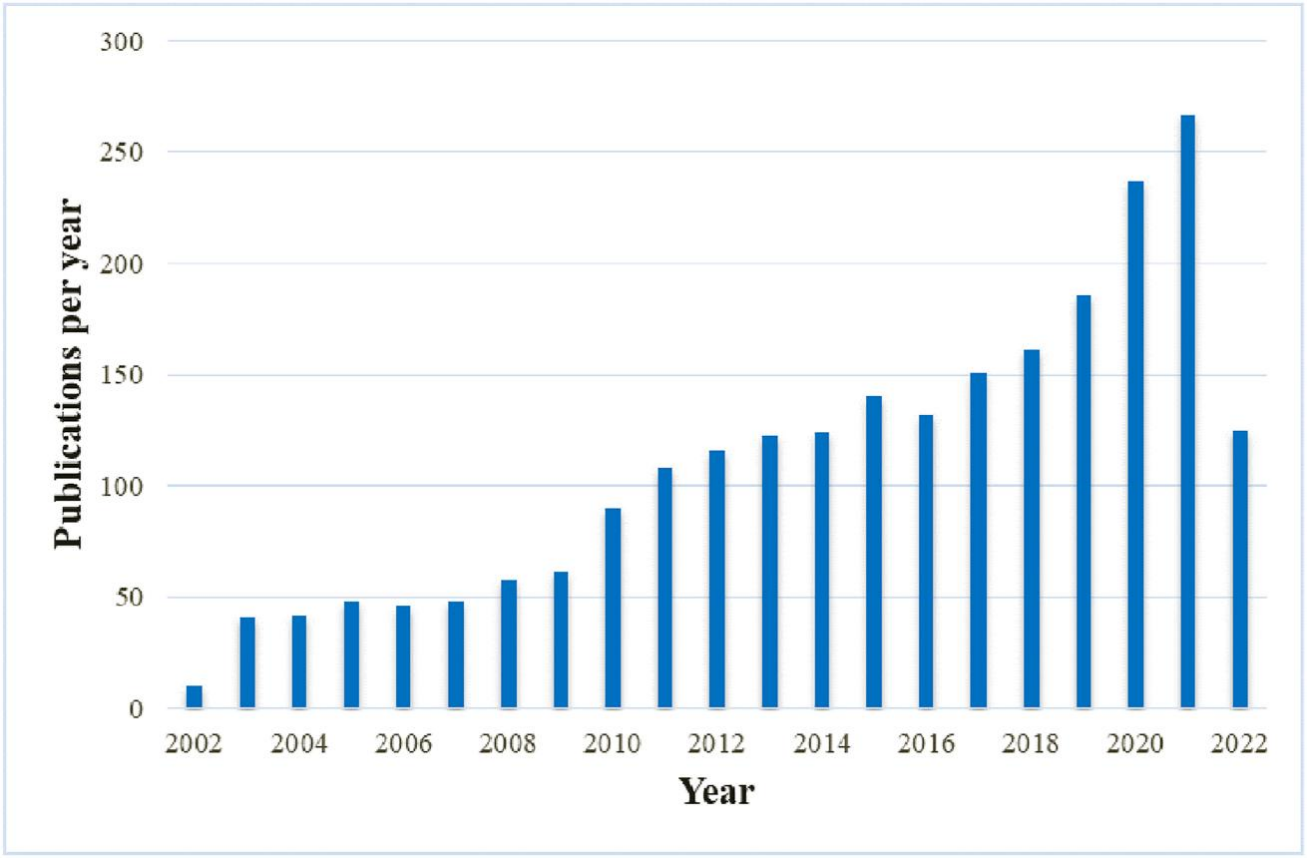
Annual number of publications about neonatal sepsis from 2002 to 2022 based on the analysis results.

As shown in Figure 2, we can intuitively find a steady upward trend of annual publications on neonatal sepsis research. The number of publications increased from 10 in 2002 to 267 in 2021. From 2002 to 2017, there was a gradual increase. In contrast, it grew rapidly between 2018 (161) and 2021 (267), demonstrating a large growth of interest in this subject of study over the previous 4 years. There were 125 studies published in 2022 in this field as of the day of the literature search; this number is probably going to rise.

### 3.3 Distribution of countries/regions

Researchers from 106 countries/regions published literature on neonatal sepsis, with the USA (556), China (224), India (194), England (155), and Turkey (140) being the top 5 countries/regions. This finding reflected the great contributions these countries/regions had made in this research field, and the USA was in a leading position in the field. The USA had the highest national centrality of 0.53, more than three times that of Australia (0.16), which ranked second, indicating strong collaborative relationships between the USA and other countries/regions (Table S1).

**FIGURE 3:**
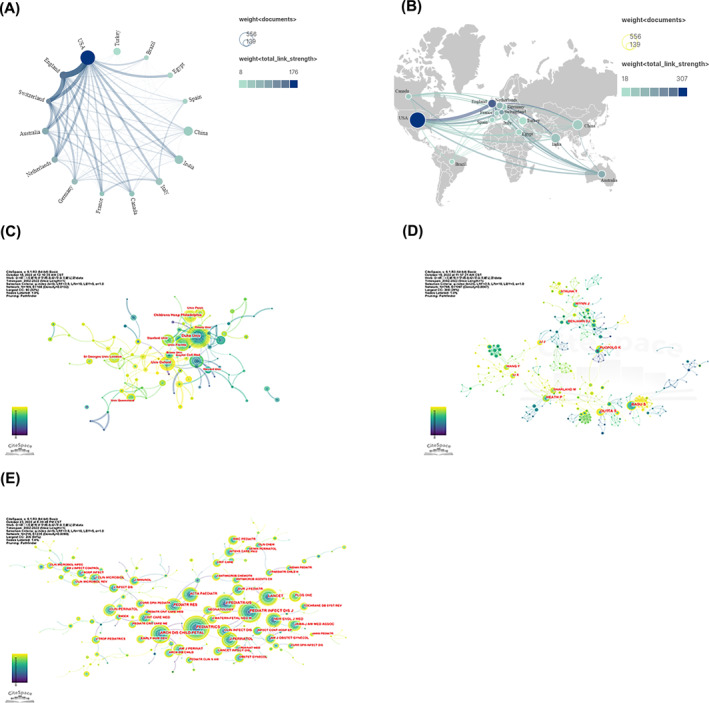
(A) Network of top 15 countries'/regions' cooperation. The thickness of lines between two countries/regions indicated the strength of cooperation, and the size and the color shade of the circles represented the number of publications and the total cooperation strength in the country/region, respectively. (B) World map showing the international cooperation among top 15 countries/regions. Each country/region is represented as a node, and each line means a coauthorship relationship. The node size is proportional to the collaboration link strength. (C) Network of institutions' cooperation on neonatal sepsis research. Each circle represents an institution. The size of the circle is positively related to the number of articles published by the organization, and the thickness of the line is positively correlated with the frequency of cooperation. (D) Network of authors' cooperation on neonatal sepsis research. Each circle represents an author. The size of the circle is positively related to the number of articles published by the author, and the thickness of the line is positively correlated with the frequency of cooperation. (E) Network of journals' cooperation on neonatal sepsis research. Each circle represents an author. The size of the circle is positively related to the number of articles published by the author, and the thickness of the line is positively correlated with the frequency of cooperation.

Figure 3A,B showed the international cooperation among the top 15 countries/regions in the number of papers published. The thickness of lines between two countries/regions indicated the strength of cooperation, and the size and the color shade of the circles represented the number of publications and the total cooperation strength in the country/region, respectively. The USA played the most important role in the cooperation between countries/regions and the USA had the closest cooperation with England and Switzerland, as shown in Figure 3A. From the world map of country/region contributions (Figure 3B), we observed that the vast majority of publications and cooperation between countries/regions were from the USA and Europe. Despite producing the second and third most publications, respectively, China and India's publications were less engaged in international cooperation.

### 3.4 Distribution of institutions

The top 10 producing institutions were listed in Table S1. Seven American and three English institutions made up the top 10 most prolific institutions. This finding highlighted the strong influence of American institutions in this area. Specifically, Duke University in the USA came in first place with 46 publications, followed by the University of Oxford (33), Children's Hospital of Philadelphia (29), and the University of Florida (29). We also analyzed the cooperative relationships of major institutions, and the results of the analysis of CiteSpace were shown in Figure 3C. None of the top 10 institutions had a centrality of more than 0.1, indicating that mutual interactions and cooperation were relatively limited.

### 3.5 Distribution of authors

A total of 10,648 authors published papers on neonatal sepsis, and Table S2 summarized the characteristics of the top 10 most prolific authors. BASU S had the greatest number of publications (24), followed by DUTTA S (23) and HEATH P (19). The centrality values of the top 10 authors were not high, and the cooperation network among authors (Figure 3D) did not show a strong cooperative link among these authors.

### 3.6 Distribution of journals

All papers were published in 528 journals, and the top 10 journals with the highest number of publications in our search were shown in Table S3. The Pediatric Infectious Disease Journal (105) was the most prolific journal, followed by the Journal of Maternal‐Fetal and Neonatal Medicine (77) and the Journal of Perinatology (69). According to the 2021 Journal Citation Reports standards, the impact factor of the top 10 journals ranged from 2.323 (Journal of Maternal‐Fetal & Neonatal Medicine) to 9.703 (Pediatrics) and was classified into Q1–Q4 categories. The results of the cooperative relationship analysis of CiteSpace were shown in Figure 3E.

### 3.7 Keywords

**FIGURE 4:**
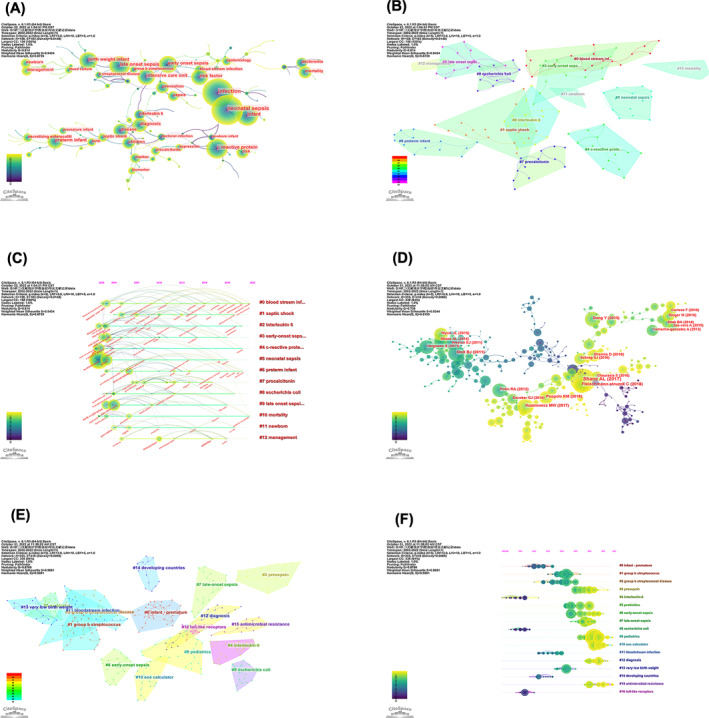
(A) Network of keyword occurrence on neonatal sepsis research. Each circle represents a keyword. The size of the circle is positively related to the frequency of the keyword, and the thickness of the line is positively correlated with the degree of association. (B) Clustered networks of keywords. Each color represents a cluster. (C) Time line view of the keywords in the field of neonatal sepsis. (D) Network of co‐cited references on neonatal sepsis. Each circle represents a citation; the size of the circle is positively related to the cited frequency, and the link between the two circles represents the two references cited in the same article. (E) The clustered network map of co‐cited references on neonatal sepsis. (F) Time line view of the clusters of citing articles in the field of neonatal sepsis.

Keywords are the highly refined content of the full text, which are used to express the subject matter of publication. Through the analysis of keywords, we can understand the research hot spots in specific fields. The top 20 keywords with the highest frequency of co‐occurrence were displayed in Table S4, and the correlation network between keywords was shown in Figure 4A. We used CiteSpace to extract the keywords and incorporated some that had the same meaning but different spellings.

To further establish the relationships among keywords, CiteSpace was also utilized to cluster the keywords and build the visual network map. Identified keywords were divided into 13 clusters: #0 blood stream infection, #1 septic shock, #2 interleukin 6, #3 early‐onset sepsis, #4 C‐reactive protein, #5 neonatal sepsis, #6 preterm infant, #7 procalcitonin, #8 *Escherichia coli*, #9 LOS, #10 mortality, #11 newborn, and #12 management (Figure 4B).

**FIGURE 5:**
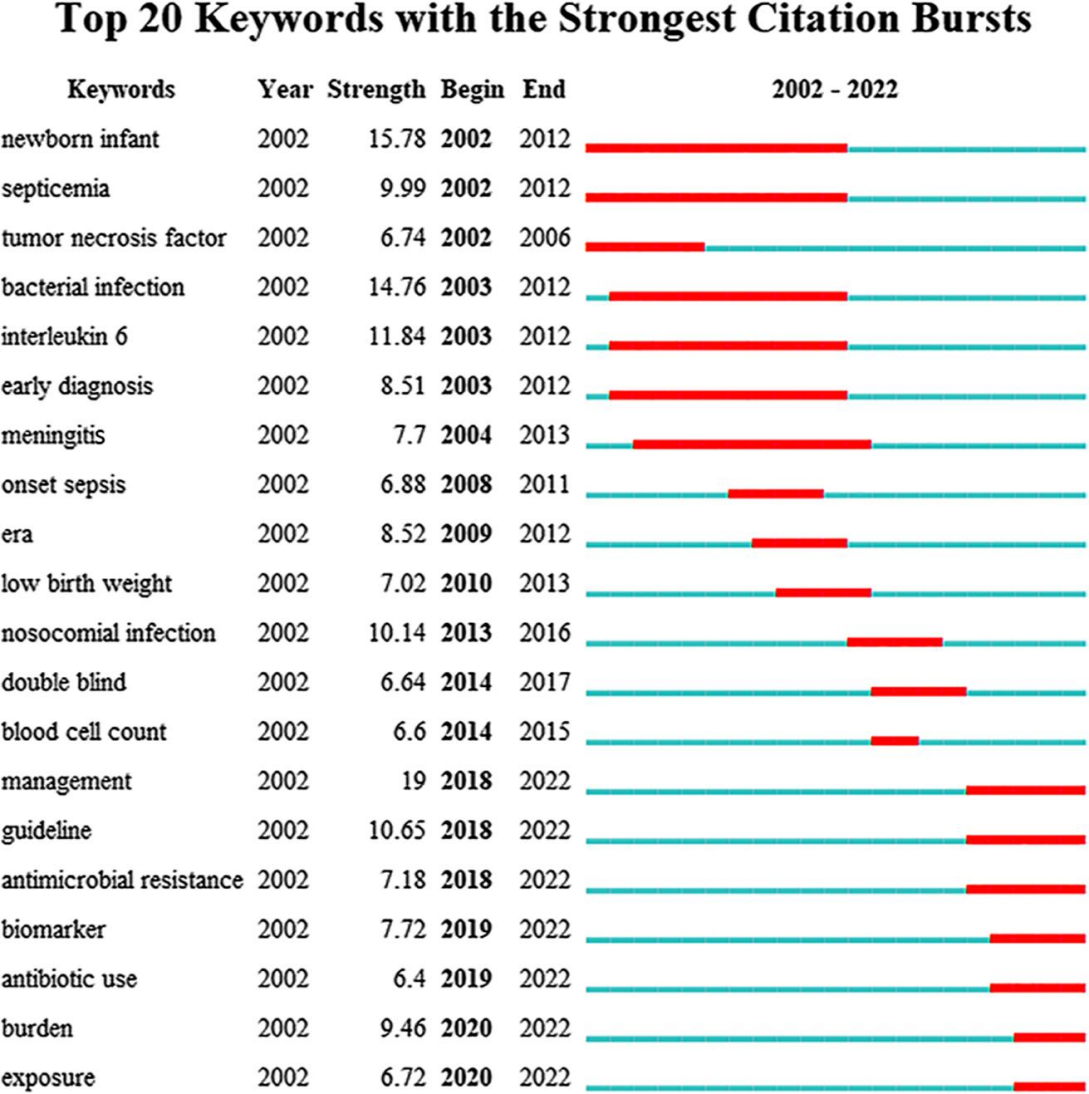
Top 20 keywords with the strongest citation bursts. The keyword marked in red indicates a sudden increase in the frequency of the keyword during this period. Keywords marked in blue indicate a period of relative unpopularity.

We also performed keyword burst analysis, a detection identifying the keywords receiving high attention from researchers in one period, through which research hot spots could be discovered. The burst strength represents the intensity of the rapid growth of a keyword. As shown in Figure 5, between 2002 and 2012, tumor necrosis factor, interleukin‐6, early diagnosis, and meningitis were the most important outburst keywords with practical significance. However, in the recent 5 years (2018–2022), management, guideline, antimicrobial resistance, biomarker, and burden have become the indicators of cutting‐edge topics, which may be future trends in neonatal sepsis research.

The time line view was used to explore the developmental path and phased hot spots in the field from the time dimension. Figure 4C displayed a time line chart of neonatal sepsis based on CiteSpace software, from which we found in similar keyword evolution trends to those shown in the burst word graph.

### 3.8 Co‐cited reference and reference burst

Co‐citation analysis indicated that two references appeared in the reference list of a third citation literature, and then, the two references formed a co‐citation relationship. The top 10 high‐cited publications on neonatal sepsis with the most citations were presented in Table S5. “Neonatal sepsis,”^8^ authored by Shane AL and published in Lancet was the most co‐cited reference on neonatal sepsis. The co‐citation and clustered network map were generated by CiteSpace from 40,662 references in a hierarchical order, and the co‐cited references were clustered into 17 major cluster labels including #0 infant‐premature, #1 Group B streptococcus (GBS), #2 group b streptococcal disease, #3 presepsin, #4 interleukin‐6, #5 probiotics, #6 early‐onset sepsis, #7 late‐onset sepsis, #8 *E. coli*, #9 pediatrics, #10 EOS calculator, #11 bloodstream infection, #12 diagnosis, #13 very low birth weight, #14 developing countries, #15 antimicrobial resistance, and #16 toll‐like receptors (Figure 4D,E).

**FIGURE 6:**
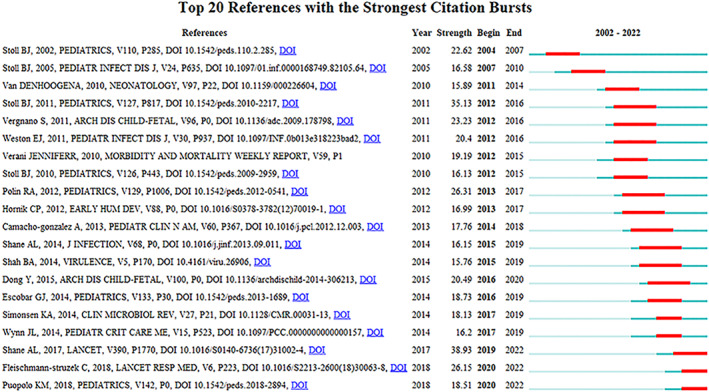
The top 20 references with the highest citation bursts. The time line marked in red indicates a sudden increase in the citation frequency during this period.

Figure 4F displayed the time line chart of distinct co‐citation, showing that cluster #9 pediatrics had the largest degree of citation bursts, and the research focus appeared to shift to antimicrobial resistance and EOS calculator. The term “citation burst” refers to references that were frequently cited over some time.^9^ Figure 6 revealed that among the top 20 references with the most robust citation bursts, the review entitled “neonatal sepsis,” the most co‐cited reference in this field as mentioned above, also had the greatest burstiness (strength = 38.93).

## 4 DISCUSSION

Bibliometric analysis is a brilliant approach for researchers to obtain systematic and visual knowledge structures and to explore and predict future trends in potential topics.^5^, ^6^ Herein, we performed a comprehensive bibliometric analysis of publications on neonatal sepsis from the WoSCC database from 2002 to 2022.

### 4.1 General information

Over the 20 years of reports on neonatal sepsis studied (2002–2022), there was an increasing annual trend of global publications, and the rapid surge after 2018 suggested that the research on neonatal sepsis had entered a mature stage and was possibly becoming a research hot spot (Figure 2). Our results revealed that the USA exhibited the highest research output and embraced vast global efforts on neonatal sepsis, placing a high value on exchanges and cooperation in the academic community. Taken together, the USA was the most influential country in this research field, far ahead of other countries/regions. Although China published the second‐highest number of publications on neonatal sepsis, transnational cooperation and information exchange between China and other countries/regions were limited. Consequently, it is urgently advised that Chinese researchers widen their international cooperation to boost their impact. Institutions and authors from high‐income countries/regions were more likely to generate high‐quality studies with close communication and cooperation with others, demonstrating that economic power had an impact on scientific research abilities. However, because of a paucity of research in poor countries/regions, the true burden of neonatal sepsis in these countries/regions might be misrepresented and underestimated.

### 4.2 Research hotspots

Keyword and citation analysis can detect research hot spots, identify emerging trends and sudden changes in disciplinary development, and highlight active or cutting‐edge research topics.^5^, ^6^ According to the results of keyword and citation analysis, we summarized three research priorities on neonatal sepsis: pathogen, diagnosis, and management.

### 4.3 Pathogen

GBS infections emerged as the primary cause of EOS in the 1970s.^10^ In 1996, the Centers for Disease Control and Prevention (CDC) developed a consensus recommending the use of intrapartum antimicrobial prophylaxis in women with risk factors,^11^ which gave rise to a substantial decline in the incidence of GBS disease among neonates in subsequent years.^12^ What's more, in 2002, it was reported that *E. coli* was overtaking GBS in EOS,^13^ and this was the possible reason why *E. coli* became a hot spot at that time, as shown in the time line chart of co‐citation analysis (Figure 4F). However, around 2010, several studies with high co‐citations indicated that despite the broad implementation of GBS prevention guidelines, GBS remained the leading cause of the remaining EOS burden.^14^, ^15^ Meanwhile, CDC updated the GBS prevention guideline^16^ in 2010 to identify missed opportunities for GBS prevention. These publications led to the hot spots of GBS disease from 2008 to 2014 in the time line chart of co‐citation analysis (Figure 4F). Of note, the above could not reflect the research status in low‐ and middle‐income countries/regions, where Gram‐negative bacteria such as *Klebsiella pneumoniae*, *Acinetobacter baumannii*, and *E. coli* are the most frequently reported causes of bacterial neonatal sepsis,^17^ and “*Klebsiella pneumonia*” became a new keyword in 2016 in the keyword time line chart (Figure 4C), which might be attributed to an increased number of publications in this field in low‐ and middle‐countries/regions.

### 4.4 Diagnosis

Diagnosis of neonatal sepsis can be difficult because the clinical and laboratory signs of neonatal sepsis are nonspecific and might mimic other noninfectious conditions.^8^ Culture is the gold standard for establishing a diagnosis of neonatal sepsis, but it often yields false negative results and has a time delay in obtaining results.^18^ Therefore, researchers and neonatologists have been striving to develop a rapid and accurate diagnostic tool for neonatal sepsis. Early diagnosis has a positive impact on reducing mortality and improving the prognosis of neonatal sepsis, and keyword burst analysis indicated “early diagnosis” was a keyword from 2003 to 2012 (Figure 5), suggesting that the primary research direction at that time was to find a marker for rapid diagnosis. The time line chart of keyword analysis demonstrated C‐reactive protein, tumor necrosis factor and interleukin‐6 were the initial research emphases, and procalcitonin enriched the original research topics in 2007 (Figure 4C). These markers contributed to make a rapid diagnosis of neonatal sepsis and have been extensively evaluated in neonates with suspected sepsis; however, their diagnostic value might be limited.^19^ As a consequence, there was a growing interest in finding novel tools with higher accuracy and sensitivity to diagnose neonatal sepsis, which was one of the current research directions related to neonatal sepsis.^20^, ^21^ As the time line chart of co‐citation analysis showed, presepsin and EOS calculator were new frontiers of research on neonatal sepsis diagnosis.

### 4.5 Management

According to keyword burst analysis, “management,” “guideline,” “antimicrobial resistance,” and “antibiotic use” have been highly cited since 2018 (Figure 5), while the co‐citation time line chart also indicated that antimicrobial resistance was an emerging topic of a subject (Figure 4F). It appeared that the management of neonatal sepsis, especially judicious use of antibiotics, had become the newest hot spot in recent years and might be a promising direction for future research. CDC has released three sets of guidelines for the management of EOS since 1996, recommending intrapartum antibiotic prophylaxis to prevent EOS related to GBS.^11^, ^16^, ^22^ Empirical treatment of neonatal sepsis typically comprises the use of aminoglycoside and β‐lactam aminopenicillin, most frequently ampicillin and gentamicin. If a culture is positive, pathogen‐directed therapy should be commenced based on sensitivities.^23^ However, antibiotic overuse due to insufficient precision of current diagnostic tools and resulting concern of missing sepsis^24^ pave the way for development of antimicrobial resistance.^25^, ^26^ A growing body of evidence has suggested an alarming upward trend in multidrug‐resistant (MDR) neonatal infections across the world,^27^, ^28^ and it is estimated that 50%–70% of common Gram‐negative isolates now are MDR.^29^ There is an urgent call for antimicrobial stewardship programs to rationalize antibiotic use and mitigate untoward consequences of antibiotic use like toxicity and the emergence of resistance.^30^

### 4.6 Limitations

The present study also has some limitations. First, we only included the publications in the WoSCC database, but publications in some other databases, such as PubMed and Scopus were omitted. Second, only English publications were enrolled, and we might underestimate the value of non‐English publications. Third, since the database is still being updated, the results of bibliometric analysis might be slightly different from the current research situation. Finally, the influence of recently published high‐quality articles might also be underestimated because they might not have enough time to accumulate sufficient citations.

## 5 CONCLUSION

In summary, we performed a comprehensive bibliometric analysis of publications on neonatal sepsis from 2002 to 2022 in this study. The USA was the leading country with the highest influence on achievements in this field. The research priorities of neonatal sepsis included pathogen, diagnosis, and management. Novel diagnostic biomarkers and judicious use of antibiotics are the hot spots receiving the most attention now and may become research directions in the future. This study brought new ideas for the studies related to neonatal sepsis and might benefit further research.

## AUTHOR CONTRIBUTIONS

**Chang Liu**: Conceptualization; data curation; formal analysis; investigation; methodology; project administration; resources; software; validation; visualization; writing – original draft preparation; writing – review & editing. **Feifan Chen**: Data curation; formal analysis; investigation; methodology; project administration; resources; software; validation; visualization; writing – original draft preparation. **Yuan Shi**: Conceptualization; funding acquisition; supervision; writing – review & editing.

## CONFLICT OF INTEREST STATEMENT

Yuan Shi is the Deputy Editor‐in‐Chief of Pediatric Discovery. To minimize bias, he was excluded from all decision‐making related to the acceptance of this article for publication. The other authors declare no conflict or interest.

## ETHICS STATEMENT

Ethical approval or individual consent is not applicable.

## PATIENT CONSENT STATEMENT

Not applicable.

## PERMISSION TO REPRODUCE MATERIAL FROM OTHER SOURCES

Not applicable.

## CLINICAL TRIAL REGISTRATION

Not applicable.

## Supporting information

Tables S1–S5

## Data Availability

Data sharing is not applicable to this article as no datasets were generated or analyzed during the current study.
