# Supplementary material for: Bibliometric analysis of neonatal sepsis from 2002 to 2022
Source: Pediatr Discov. 2024 Mar 15;2(1):e49. doi: 10.1002/pdi3.49 (PMC12118236; doi:10.1002/pdi3.49)
Supplement: Supplementary file 1 — Tables S1–S5 [file PDI3-2-e49-s001.docx]

| Rank | Country /Region | Papers | Centrality | Institution | Country /Region | Papers | Centrality |
| --- | --- | --- | --- | --- | --- | --- | --- |
| 1 | USA | 556 | 0.53 | Duke University | USA | 46 | 0.05 |
| 2 | China | 224 | 0.06 | University of Oxford | England | 33 | 0.08 |
| 3 | India | 194 | 0.05 | Children's Hospital of Philadelphia | USA | 29 | 0.02 |
| 4 | England | 155 | 0.13 | University of Florida | USA | 29 | 0.05 |
| 5 | Turkey | 140 | 0.01 | University of Pennsylvania | USA | 28 | 0 |
| 6 | Italy | 129 | 0.06 | Baylor College of Medicine | USA | 24 | 0.02 |
| 7 | Australia | 122 | 0.16 | St George's, University of London | England | 21 | 0.09 |
| 8 | Netherlands | 102 | 0.10 | Brown University | USA | 20 | 0.06 |
| 9 | Germany | 98 | 0.02 | University College London | England | 20 | 0.02 |
| 10 | Egypt | 83 | 0.01 | Emory University | USA | 19 | 0 |

Supplementary Table 1. The top 10 countries/regions and institutions of publications on neonatal sepsis from 2002 to 2022.

| Rank | Author | Papers | Centrality | Institution | Country |
| --- | --- | --- | --- | --- | --- |
| 1 | BASU S | 24 | 0.10 | National Institute of Cholera and Enteric Diseases | India |
| 2 | DUTTA S | 23 | 0.09 | McMaster University Children's Hospital | Canada |
| 3 | HEATH P | 19 | 0.06 | Health Protection Agency Centre for Infections | England |
| 4 | PUOPOLO K | 18 | 0.07 | Children's Hospital of Philadelphia | USA |
| 5 | WYNN J | 17 | 0.05 | University of Florida | USA |
| 6 | SHARLAND M | 15 | 0.02 | St George's, University of London | England |
| 7 | WANG Y | 14 | 0.05 | Capital Medical University | China |
| 7 | LI J | 14 | 0.04 | Sichuan University | China |
| 7 | LI X | 14 | 0.01 | Maternity and Child Health Care of Zaozhuang | China |
| 7 | STRUNK T | 14 | 0.01 | University of Western Australia | Australia |

Supplementary Table 2. The top 10 authors on publications of neonatal sepsis from 2002 to 2022.

| Rank | Journal | Papers | Impact factor (2021) | Quartile in category |
| --- | --- | --- | --- | --- |
| 1 | Pediatric infectious disease journal | 105 | 3.806 | Q4 |
| 2 | Journal of maternal-fetal & neonatal medicine | 77 | 2.323 | Q4 |
| 3 | Journal of perinatology | 69 | 3.225 | Q3 |
| 4 | Plos one | 61 | 3.752 | Q3 |
| 5 | American journal of perinatology | 60 | 3.079 | Q4 |
| 6 | Acta paediatrica | 55 | 4.056 | Q4 |
| 7 | Pediatric research | 54 | 3.953 | Q3 |
| 8 | Pediatrics | 42 | 9.703 | Q2 |
| 9 | Frontiers in pediatrics | 39 | 3.569 | Q3 |
| 10 | Archives of disease in childhood-fetal and neonatal edition | 38 | 6.643 | Q1 |

Supplementary Table 3. The top 10 journals on publications of neonatal sepsis from 2002 to 2022.

| Rank | Keyword | Papers | Rank | Keyword | Papers |
| --- | --- | --- | --- | --- | --- |
| 1 | neonatal sepsis | 677 | 11 | intensive care unit | 207 |
| 2 | infection | 607 | 12 | diagnosis | 200 |
| 3 | infant | 313 | 13 | risk | 161 |
| 4 | c reactive protein | 311 | 14 | mortality | 161 |
| 5 | late onset sepsis | 291 | 15 | disease | 147 |
| 6 | preterm infant | 285 | 16 | management | 144 |
| 7 | early onset sepsis | 240 | 17 | children | 133 |
| 8 | birth weight infant | 231 | 18 | prevention | 132 |
| 9 | newborn | 221 | 19 | necrotizing enterocolitis | 113 |
| 10 | risk factor | 208 | 20 | bacteremia | 104 |

Supplementary Table 4. The top 20 keywords on publications of neonatal sepsis from 2002 to 2022.

| Rank | Title | Journal | Year | Author | Citations |
| --- | --- | --- | --- | --- | --- |
| 1 | Neonatal sepsis | LANCET | 2017 | Shane AL | 176 |
| 2 | The global burden of paediatric and neonatal sepsis: a systematic review | LANCET RESP MED | 2018 | Fleischmann-struzek C | 93 |
| 3 | A Quantitative, Risk-Based Approach to the Management of Neonatal Early-Onset Sepsis | JAMA PEDIATR | 2017 | Kuzniewicz MW | 78 |
| 4 | Early Onset Neonatal Sepsis: The Burden of Group B Streptococcal and E. coli Disease Continues | PEDIATRICS | 2011 | Stoll BJ | 75 |
| 5 | Management of Neonates Born at ≥35 0/7 Weeks’ Gestation With Suspected or Proven Early-Onset Bacterial Sepsis | PEDIATRICS | 2018 | Puopolo KM | 67 |
| 6 | Late-onset neonatal sepsis: recent developments | ARCH DIS CHILD-FETAL | 2015 | Dong Y | 67 |
| 7 | Management of Neonates With Suspected or Proven Early-Onset Bacterial Sepsis | PEDIATRICS | 2012 | Polin RA | 60 |
| 8 | Defining neonatal sepsis | CURR OPIN PEDIATR | 2016 | Wynn JL | 55 |
| 9 | Biomarkers for diagnosis of neonatal sepsis: a literature review | J MATERN-FETAL NEO M | 2018 | Sharma D | 54 |
| 10 | Neonatal infections in England: the NeonIN surveillance network | ARCH DIS CHILD-FETAL | 2011 | Vergnano S | 50 |

Supplementary Table 5. The top 10 high-cited publications of neonatal sepsis from 2002 to 2022.
